# The need for masked genomes in gymnosperms

**DOI:** 10.3389/fpls.2023.1309744

**Published:** 2023-12-07

**Authors:** Pengkai Zhu, Tianyou He, Yushan Zheng, Lingyan Chen

**Affiliations:** Fujian Agriculture and Forestry University, Fuzhou, China

**Keywords:** gymnosperm, ultra-large genome, repeats, masked genome, sequencing read mapping

## 1 Introduction

With the rapid advancement of Next-Generation Sequencing (NGS) technologies, researchers have gained access to an ever-increasing amount of biological genome data, including many ultra-large genomes (>10 Gb). These ultra-large genomes are typically found in species of gymnosperms, excluding the gnetophytes (Wan et al., 2022). Examples of such species include *Cycas* (10.5 Gb) (Liu et al., 2022), *Ginkgo* (10.6 Gb) (Liu et al., 2021), Norway spruce (12.3 Gb) (Nystedt et al., 2013), Chinese pine (25.4 Gb) (Niu et al., 2022), and coast redwood (26.5 Gb) (Scott et al., 2016). However, due to the inherently large genomes of gymnosperms, conducting whole-genome studies has become exceedingly challenging (Wang and Ingvarsson, 2023). Handling these vast genomic datasets typically demands substantial computational resources. Ultra-large genomes in gymnosperms are characterized by an abundance of repetitive sequences, chimeric genes, and intricate structural variations (Nystedt et al., 2013; Wan et al., 2022), rendering existing alignment tools often ineffective in efficiently mapping sequencing reads to these extensive genomes. Given the complexity of ultra-large genomes, the memory requirements for generating alignment indices far exceed the capabilities of many research laboratories and researchers. This has posed formidable obstacles for investigators attempting to address biological questions within the context of these ultra-large genomes.

Coniferous trees hold considerable ecological and economic value, prompting extensive researches into their entire genomes (Neale et al., 2022). Gymnosperms possess distinct genes with unique functions (Ausin et al., 2016), indicating their high potential for functional genomics studies. In order to study gene function in gymnosperms, identifying differentially expressed genes is a primary avenue for exploring functional genes (Costa-Silva et al., 2017), with RNA-seq serving as an indispensable tool for analyzing differential gene expression at the transcriptome level (Stark et al., 2019). Moreover, software tools such as STAR (Dobin et al., 2013) and HISAT2 (Kim et al., 2019) have demonstrated high accuracy and sensitivity in detecting specific transcripts and certain genes when mapping RNA-seq reads to the genome (Sahraeian et al., 2017). However, prior to mapping, the process of indexing genome often consumes a substantial amount of physical memory. For instance, HISAT2 constructs a reference genome index for the 3GB human genome with structure annotation file, it can peak at a memory consumption of up to 200GB (Kim et al., 2019). As genome sizes increase, the memory requirements escalate accordingly. Notably, *Paris japonica* with a genome size of 145 Gb (Pellicer et al., 2010), represents the largest genomes among eukaryotes, presenting a formidable challenge when it comes to handling large genome alignment tasks that demand significant physical memory.

In light of this, we suggest that when publishing the genomes of gymnosperms, researchers should also provide a version of the genome where repeat regions have been masked or at least provide an annotation file for repeat regions to facilitate their use by other researchers.

## 2 Alignment strategies for sequencing reads in gymnosperms

In order to effectively map sequencing data to the reference genome for gene function studies in gymnosperms, we have explored some strategies to address the challenges. The most straightforward approach involves increasing physical memory space, but this often entails expensive hardware upgrades. Alternatively, in some special cases, such as when aligning RNA-seq sequencing data, we can employ a reference-free method to handle the sequencing data. However, due to the absence of a reference genome for validation, reference-free analysis may lead to a higher incidence of false positives, where genes or transcripts are erroneously identified as present when they do not actually exist (Lee et al., 2021). Therefore, the optimal scenario is to align the sequencing reads to the index established by the hard-masked genome, reducing memory usage without altering the sequence and positional information of the genes. To assess the effect of using a hard-masked genome in build indices, we employed the STAR (v2.7.8, parameters: –runThreadN 20 –runMode genomeGenerate) to build index for the longest chromosomes of four gymnosperm species. Our findings reveal a notable reduction in maximum resident set size (i.e., the peak memory usage) when building indices using a hard-masked genome (**Table 1**). Apart from the repeat regions, all other regions are considered as effective areas for index building. Consequently, species with a higher proportion of repeat sequences might exhibit a more pronounced reduction in maximum resident set size during index building. However, this observation is not an absolute rule, as the memory utilization during index creation is also influenced by the complexity of gene structure annotation.

**Table 1:** Comparison of peak memory usage in unmasked and repeat-masked sequences in the longest chromosomes of four gymnosperm species.

Species	Longest Chromosome Size	Repeats Proportion	Maximum Resident Set Size (kbytes)		Repeat-Masked Memory Proportion
			Unmasked	Repeat-masked	
*Welwitschia mirabilis*	551,969,684	44.40%	13,327,256	8,150,212	61.15%
*Cycas panzhihuaensis*	692,804,514	54.56%	17,606,716	8,851,680	50.27%
*Ginkgo biloba*	1,185,857,400	45.52%	15,289,608	9,292,320	60.78%
*Pinus tabuliformis*	1,275,696,759	57.82%	26,254,340	15,499,500	59.04%

The large sizes of gymnosperm genomes are primarily attributed to the historical and ongoing activity of retrotransposons or transposable elements (TEs), including long terminal repeat (LTR) retrotransposons (Feschotte et al., 2002; Lim et al., 2007). Additionally, in gymnosperms, species with larger genomes tend to have a higher proportion of intact LTRs and a lower proportion of solo LTRs compared to species with smaller genomes (Moffat, 2000; Nystedt et al., 2013; Wan et al., 2018; Xiong et al., 2021; Niu et al., 2022). Therefore, by masking repeat regions in the genome, we can reduce the physical memory usage during the index building process.

## 3 Impact on masked gymnosperm genome alignment

Compared to angiosperms, gymnosperms, particularly those with large genomes, are more prone to annotation issues due to a higher proportion of repetitive sequences (Zhang et al., 2023). The presence of repeats can lead to misinterpretation of gene boundaries, affecting the accuracy of gene annotations (Nystedt et al., 2013; Wang and Ingvarsson, 2023). Furthermore, in automated annotation pipelines, tools like BRAKER (Hoff et al., 2019) and MAKER (Campbell et al., 2014) are frequently employed to utilize repeat-masked genome for annotation. This practice aims to avoid noise and reduce computational burden (Pham et al., 2020; Mei et al., 2021; Yang et al., 2022). It’s worth noting that in some specific studies, only a small part of the genome is focused on. For example, in RNA-seq read mapping, researchers primarily focus on transcript regions (Conesa et al., 2016). Therefore, when researchers aim for regions that have been annotated in the genome, mapping sequencing reads to the masked genome may not have a significant impact on the results or outcomes of the specific studies.

Gymnosperms are characterized by the presence of exceptionally long genes (Nystedt et al., 2013; Guan et al., 2016; Niu et al., 2022), often distinguished by their very long introns (Wan et al., 2021). These introns sometimes incorporate LTRs and TEs (Stival Sena et al., 2014). In Chinese pine, it appears that long introns do not significantly affect transcription accuracy (Niu et al., 2022). However, long genes in *Picea glauca* and *Picea tabuliformis* tend to exhibit higher expression levels (Ren et al., 2006; Stival Sena et al., 2014). Conversely, some studies in other organisms have suggested that compact genes often display higher expression levels (Castillo-Davis et al., 2002; Stenøien, 2007). The confusion regarding the correlation between gene length and expression level may be attributed to the overrepresented reads from long transcripts, leading to statistical biases in RNA sequencing data (Project et al., 2013; Wan et al., 2022). Therefore, genome-wide masking of repeats has the potential to reduce intron length to some extent while preserving gene structure, thus effectively minimizing alignment errors.

## 4 Convenient workflow for obtaining masked genomes

To assist researchers in more easily obtaining a masked genome of ultra-large genome species for genome alignment, we have organized a workflow. It provides researchers with instructions on creating a masked genome either through sequence-based masking or via the annotation of repeat regions within the sequence.

Here are the main steps of this workflow: In the scenario where only the genome sequence file is available, there is a need to perform repeat sequence prediction and masking operations. Firstly, employ Red (Girgis, 2015) to predict repetitive sequences within the genome. Subsequently, transform the genome that has been software-masked into a hard-masked genome. In the situation where both the genome sequence file and repeat annotation are provided, the provided repeat annotation file can be converted into a bed file. Then, utilize the BEDTools (Quinlan and Hall, 2010) to mask repeat regions based on the information within the bed file.

This method provides researchers with a more flexible solution. The code and resources related to this workflow can be found in the GitHub repository (https://github.com/pk-zhu/APMG), for use and further improvement by the scientific community.

## 5 Discussion

Using a repeat-masked genome for alignment, particularly in gymnosperms where genome expansion has been driven by an increase in the proportion of repeats (Wan et al., 2022), can lead to reduced memory usage during the indexing of a genome for downstream analysis. However, this approach may present some potential drawbacks in accurately representing genomic complexity. Firstly, since more sequences are masked in gymnosperms, alignment inaccuracies may occur during whole-genome alignment. These inaccuracies might manifest when using a masked genome for read mapping due to the absence of certain information. For example, some reads might align to both ends of the masked region due to the absence of nucleotides in the sequence, resulting in discrepancies in downstream analyses reliant on alignment, such as estimating transcript abundance. However, this possibility can be mitigated by setting a stringent mismatch tolerance. Furthermore, due to the masking of certain sequences, some genuinely multi-mapped reads may be considered as uniquely mapped reads. However, since the prediction of repeats is based on sequence similarity, only a limited number of reads might experience this situation, and this inaccuracy will be diminished with updates in repeat sequence prediction algorithms. Additionally, for variant calling analysis based on alignment results, masking repeat regions can effectively eliminate false duplicates of a set of related genes, thereby enhancing the accuracy of variant detection (Catreux et al., 2021; Wagner et al., 2022). Given the substantial presence of repeats in gymnosperm genomes, using a masked genome might therefore yield more accurate results.

However, reads generated from whole-genome sequencing techniques such as ATAC-seq (Buenrostro et al., 2013), BS-seq (Krueger et al., 2012), and RAD-seq (Davey and Blaxter, 2011) are not recommended to be used with a masked genome in analysis. These techniques are commonly employed for sequencing across the entire genome, aiming to comprehend the structure, functionality, and variations within the genome. Therefore, these kinds of data might be required for alignment across the whole genome or for computing abundance signals of reads aligned to repeat regions. Additionally, DNA methylation levels are positively correlated with genome size (Novák et al., 2020), and some repeat sequences contribute to the formation of highly methylated heterochromatin (Islam-Faridi et al., 2007; Fedoroff, 2012), which is one of the critical factors for the proper expression of long genes in gymnosperms (Fuchs et al., 2008; Niu et al., 2022). Therefore, data capable of detecting methylation should also not be mapped to the repeat-masked genome.

In conclusion, when adopting the masked genome for analysis, researchers need to carefully balance the reduction in computational resource consumption with the potential loss of genetic information. It’s essential to consider this balance in the context of their research objectives and specific subjects under study. Masked genome offers a flexible and efficient solution for studying gymnosperms but should be used judiciously considering its limitations in specific research scenarios.

## Author contributions

PZ: Methodology, Writing – original draft, Writing – review & editing. TH: Funding acquisition, Writing – review & editing. YZ: Resources, Writing – review & editing. LC: Supervision, Writing – review & editing.
